# Assessment of SADC Countries’ National Adaptation Planning Health Impacts Inclusion: A Thorough Review

**DOI:** 10.5334/aogh.4458

**Published:** 2024-09-18

**Authors:** Renate Meyer, Caradee Wright, Hanna-Andrea Rother

**Affiliations:** 1 Division of Environmental Health, School of Public Health, University of Cape Town, South Africa; 2 Environment and Health Research Unit, South African Medical Research Council, Pretoria, South Africa; 3 Department of Geography, Geoinformatics and Meteorology, University of Pretoria, Pretoria, South Africa

**Keywords:** climate change, environmental health, monitoring and evaluation, policy, vulnerability

## Abstract

*Background:* The impacts of climate change are recognised as a key challenge of the 21st century. By 2030, Sub-Saharan Africa is projected to have the globally highest burden of disease due to climate change.

*Objectives:* This study aims to evaluate the strengths and weaknesses of the National Adaptation Plans (NAPs) of the Southern African Development Community (SADC), a sub-region under-represented at a global level, in addressing current and future climate change–related health impacts. It specifically assesses the NAPs of Botswana, Mozambique, Namibia, South Africa, and Zimbabwe.

*Methods:* A thorough review was conducted, analysing articles, government reports, and national communications related to NAPs and climate change health outcomes in the selected countries. Sources were evaluated against pre-defined inclusion and exclusion criteria.

*Main findings:* All five countries prioritised health in their NAPs; however, health departments were excluded from assessments in two of the countries. Although health surveillance and early warning systems were included in the NAPs, there was limited evidence of their integration into broader climate, health, economic, and labour policies. National climate change focal points were identified, but governance and implementation at district and local levels were not well-documented. This review highlighted a need for greater inclusion of Indigenous and locally led knowledge. Common barriers identified included the lack of data with appropriate frequency and scale. Governance and implementation difficulties were also identified in all five countries; these difficulties included both a lack of coordination and a lack of institutional capacity. These challenges, especially a lack of political will to address the compound impacts of altered climate and health on all earth systems, are also found at the regional level.

*Conclusions:* National strategies and implementation programs in SADC countries need to be agile in their ability to scale and adapt, yet they also need to include measurable actions and timeframes. Given the shared climate and health trends and the interconnected socio-economic, environmental, and political landscape, there is significant potential for regional coordination to address cross-border climate change impacts and to optimise resource use.

## Introduction

Environmental factors currently contribute to 24% of the annual global disease burden, a figure expected to rise over the coming decades with the increased frequency and intensity of climate hazards [1]. Over the last 20 years, there has been a 46% global increase of extreme weather events, which have contributed to increasing levels of food insecurity, poverty, and mental health issues and reducing access to education and healthcare [2–4]. This vulnerability is echoed in the Intergovernmental Panel on Climate Change (IPCC) assessment reports, which indicate that sub-Saharan Africa is projected to have the greatest burden of mortality impacts attributable to climate change by 2030 [5].

Challenges related to the development and implementation of climate-adaptation strategies are particularly evident within countries, such as those in the Southern African sub-region, that show a high vulnerability and a low adaptive capacity to the impacts of climate variability. The sub-region is defined as the total geographic area occupied by the 16 African member states of the SADC, many of which have undiversified economies, regional instability, extreme poverty, and the highest prevalence of HIV in the world [6]. In the near-term, the SADC sub-region is expected to experience an increase in extreme weather events such as heatwaves, wildfires, and floods, resulting in increased health risks. In one example, recent estimates suggest that more than 40% of heat-related deaths in South Africa can be attributed to human-induced climate change [5].

More than half the countries in the SADC sub-region are ranked as the most inequitable in the world [7]. Barriers to adaptation to climate change include infrastructure backlogs, institutional weaknesses, low education and skills capacity, and high rates of poverty and inequality [8]. These vulnerabilities and developmental challenges are heightened by multiple stressors such as conflict and high levels of gender and economic disparity [9].

Such disparity—coupled with economic, social, and legal barriers (including governance and political issues)—hinder regional development and collaboration. Without substantial climate and development action in the region within the next 30 years, more than 86 million people could be internally displaced due to slow-onset impacts, including water scarcity, low crop productivity, and a rise in sea level [10]. The heavy reliance on climate-sensitive economic sectors and low resilience further limit the region’s capacity to adapt. These barriers also affect the ability to implement NAPs and Health National Adaptation Plans (H-NAPs), which are crucial for building resilience in the face of climate-related health impacts.

## International and Regional Treaties and Agreements

Under the UNFCCC mandate, member states submit Nationally Determined Contributions (NDCs) outlining actions to meet the Paris Agreement goals. Despite challenges, the UNFCCC remains central in holding states accountable [11–14]. The NAPs outline the country-level actions needed to meet those goals, and members are encouraged to include health indicators in those plans and to subsequently develop H-NAPs [15]. While support and guidance are provided, the NAP Global Network has indicated that challenges exist concerning countries’ specific climate adaptations, accountability, and communications [16]. With the aim of creating a stronger health focus on climate ambitions, in 2021 the World Health Organization (WHO) launched the flagship Health Programme at the annual Conference of the Parties.

The African Union (AU) has supported global and regional climate responses, with all SADC members listed as signatories to the UNFCCC. At the regional level, SADC’s Health Protocol and subsequent frameworks aim to address key health issues but remain limited, with large gaps identified in both the understanding of impact pathways and the resources needed to implement adaptation programmes [17, 18].

Key to these adaptation initiatives is the access and availability of international financial support. Although estimates of the economic cost of health impacts related to climate change vary (and are based on outdated projections), the average financial inflow to the SADC region, between 2013 and 2017, was 804 million USD/year [8, 19]. During this period, Tanzania, South Africa, and Mozambique were the primary recipients of public climate funding, with more than 60% of climate finance targeting energy, water, and transport infrastructure, while less than 11% was allocated to social services, including health and education sectors [8].

## Method

A thorough review evaluated the NAPs of five SADC countries: Botswana, Mozambique, Namibia, South Africa, and Zimbabwe. The selected countries’ spatial proximity afforded the opportunity to (a) examine the NAPs’ efficacy in enabling planned implementation, (b) illustrate overlaps in climate and health impacts between neighbouring countries, and (c) analyse whether cross-border intergovernmental collaboration has (or could be) implemented to assist in adaptation efforts regarding climate change and health.

Sources included peer-reviewed studies from PubMed, EBSCOhost, Scopus, and Web of Science, as well as references from government reports and national communications. Initially, 255 records were screened, with 10 undergoing full-text analysis. Additionally, 63 studies from other sources were included in the data-extraction chart. The search was geographically restricted to the five SADC countries. Details are outlined in Supplementary material Table A.

Given the various stages of NAP development in the selected countries, the review also included international reports and guidelines from UNFCCC, NAP Global Network, WHO, UNEP, and relevant government publications. These reports are listed in the supplementary material, with data available on Figshare (ZivaHub) [20].

The review considered the UNFCCC COP17 resolution, which, with input from the Least Developed Countries expert group, provided guidance on NAP development [21]. This includes principles such as a participatory approach, gender sensitivity, and integration of traditional knowledge [22]. It is also useful to note that there are various stages in NAP development, such as National Adaptation Programmes of Action (NAPA), NAP Roadmaps, and NAP Frameworks.

Findings from the WHO 2021 Health and Climate Change Global Survey [23], (which included Mozambique, South Africa, and Zimbabwe) were consolidated in Supplementary material Table C. The full review protocol is available on ZivaHub (Figshare) [24].

## Results

Tables

- Table 1: Documentation availability by country
- Table 2: Climate change and health impacts
- Tables 3–7: Detailed country-level data

**Table 1:** Climate Change and National Health Documentation Available Online as of January 2022.

COUNTRY	NAP ADDRESSES CC AND HEALTH	H-NAP ADDRESSES CC AND HEALTH	NATIONAL ADAPTATION POLICY THAT INCLUDES HEALTH	UNFCCC NATIONAL COMMUNICATION (NC) AND NDC	NATIONAL ADAPTATION STRATEGIES THAT INCLUDE HEALTH	ADAPTATION FINANCING MENTIONED
Botswana	NAP Framework (2020)	-	Draft Climate Change Response Policy (2016)	First NC (2001) Second NC (2013) Third NC (2019) NDC (2016)	National Climate Change Strategy and Action Plan (2018)	-
Mozambique	National Adaptation Programme of Action. (2007)	-	IP	Initial NC (2006) NDC (2021)	National Climate Change Adaptation and Mitigation Strategy 2013–2025. (2012)	-
Namibia	IP	-	National Climate Change Policy (2011)	First NC (2001) Second NC (2011) Third NC (2015) Fourth NC (2020) NDC (2021)	National Climate Change Strategy and Action Plan 2013–2020	USD 82 Million needed for health
South Africa	National Climate Change Adaptation Strategy (2021)	National Climate Change and Health Adaptation Plan. 2014–2019	National Climate Change Response Policy (2011)	First NC (2003) Second NC (2011) Third NC (2018) NDC (2021)	National Climate Change Adaptation Strategy (2021)	USD 4 Billion – 380 billion (scenario dependant health not defined)
Zimbabwe	NAP Roadmap (2019)	IP	National Climate Policy (2017)	First NC (1998) Second NC (2013) Third NC (2017) NDC (2021)	National Climate Change Response Strategy (2014)	USD 52 million needed for health

KEY: Climate change (CC); Health Adaptation Plan (H-NAP); National Communication (NC); Nationally Determined Contributions (NDC); Documentation found online (Y); Documentation in Progress (IP); No data found in documents (-).

**Table 2:** Overview of Included NAPs, Strategies, and Academic Studies that List Climate Change and Health Impacts.

**CLIMATE CHANGE RISK**	**HEALTH IMPACT**	**Botswana**			**Namibia**			**Mozambique**			**South Africa**			**Zimbabwe**		
		NAP	Study Ref	National Strategy	NAP	Study Ref	National Strategy	NAP	Study Ref	National Strategy	NAP	Study Ref	National Strategy	NAP	Study Ref	National Strategy
**INCREASE IN TEMPERATURE WHICH CONTRIBUTE TO DROUGHT**	**FOOD AND WATER SECURITY**	Y	[25, 26]	Y	-	[25]	Y	Y	[27, 28]	Y	Y	[29–33]	Y	Y	[25]	Y
**INCREASE IN PRECIPITATION WHICH CONTRIBUTE TO FLOODING**	**WATER AND VECTOR-BORNE DISEASES**	Y	N	Y	-	N	Y	Y	[27, 28]	Y	Y	N	Y	Y	N	Y
**INCREASE IN TEMPERATURE**	**HEAT RELATED DISEASES**	N	N	N	-	N	Y	N	N	N	Y	[33, 24]	Y	N	N	N
**EMISSIONS AND AIR POLLUTION**	**RESPIRATORY AND CARDIO DISEASE**	N	N	N	-	N	N	N	N	N	Y	[33, 34]	Y	N	N	N
**INCREASE IN PRECIPITATION WHICH CONTRIBUTE TO FLOODING**	**PHYSICAL INJURY AND DISPLACE-MENT**	N	N	N	-	N	N	N	[27]	N	Y	[28]	Y	N	N	N
**INDIRECT EFFECTS OF EXTREME WEATHER EVENTS**	**HEIGHTENED MENTAL STRESS AND DISEASE**	N	N	N	-	N	N	N	[27]	N	N	[33, 34]	Y	N	N	N

KEY: Documentation found online (Y), No documentation found online (N), No data available (-).

**Table 3:** Country-Level Data for Botswana Collated from the Full Data-Extraction Table [20].

CLIMATE RISK	HEALTH IMPACT	NAP ADDRESSES HEALTH EXPOSURE	H-NAP ADDRESSES HEALTH EXPOSURE	IMPLEMENTATION PLANS AND MONITORING MENTIONED	INTERSECTORAL POLICY AND IMPLEMENTATION
Botswana is a water scarce country and is already facing the negative impacts of climate change as evidenced by the endemic droughts, heavy rainfall, heat waves, and severe thunderstorms.	Impacts of climate change on health will exacerbate the incidence of malaria, cholera and an increase in malnutrition. The diseases Dengue Fever, Rickettsia, Cholera, Yellow Fever, and Bilharzia may potentially be affected by changes in temperature and water availability, as may be respiratory diseases (such as asthma) and heat stress (NC3)	NAP Framework 2020. Key sectors consulted in national consultation (Health Department not included p 10) Intention to conduct national health impact assessments to provide valuable information on population vulnerability Plan to establish District Climate Change Committees that will link the subnational with the national level (p 13) Botswana’s Ministry of Health and Wellness is currently not implementing projects or programmes on health adaptation to climate change (NC3)	Need for Botswana National Health Adaptation Strategy and Action Plan as adjunct to National Climate Change Strategy to be adopted and implemented as a public health approach to adaptation to climate change (NC3)	2018 National Climate Change Strategy and Action Plan completed and awaiting approval (emphasizes the use of Indigenous and Traditional Knowledge and practice p 14). Assess the status of health facilities preparedness in handling casualties from climate change events including the ability to collaborate with local community structures and groups to enable protection of the severely injured and disabled by mental disorder Integrated environment and health surveillance Revise Public Health Act and National Health Plan to include climate change The implementation of a voluntary community-based monitoring and response system to identify community members most vulnerable to health impacts from climate change [26] National Monitoring and Evaluation System (NMES): monitor and evaluate National Development Plan 11.	Botswana Climate Change Response Policy draft 2016 Botswana Vision 2036 National Development Plan 11 include climate change threat. National Health Policy 2011 identifies climate change as a real threat to population well-being (no implementation) Revised Rural Development Policy 2002 National Disaster Risk Reduction Policy Community based Natural Resources Management policy 2007 National Conservation Strategy 2019 draft Policies on energy, climate change, waste management, and Integrated Transport Policy
Botswana is a water scarce country. The national cattle herd has suffered mortalities of one-third at least four times in the past 70 years because of drought Crop agriculture is a marginal activity but could become more precarious, particularly for subsistence farmers who are reliant on the land (NC3)	Food and water security as a risk to nutrition, sanitation, and hygiene	Establish water sanitation and hygiene committees in the Districts, Sub-districts, and Villages. Guided by the local DHMTs to plan and coordinate key activities related to the prevention and control sanitation/hygiene and water-related diseases (p 146) Develop a set of interventions required to protect nutrition from climate related risks		A number of adaptation strategies to address impacts of climate change on health are focused on agriculture and food security; maternal and child-care and feeding practices; environmental health, water, and sanitation (NC2019)	It is important that a national vulnerability assessment and adaptation process is undertaken in order to identify high risk health districts and locations and vulnerable groups
Impacts of extreme weather events and related increase in water- and vector-borne diseases	The population exposed to malaria is projected to double, and increases in a variety of other climatically linked diseases are possible	For malaria, a variety of vector control techniques are being used and may need to be expanded For diarrhoea, the strategy could include upgrading of drinking water supplies and sanitation, improved drainage and poverty alleviation		Education and awareness around health and climate change The integration of climate change related surveillance and tracking Water quality surveillance – Rolling out water sampling plans to district and villages to trace and detect early contaminants	

**Table 4:** Country-Level Data for Mozambique Collated from the Full Data-Extraction Table [20].

CLIMATE	HEALTH	NAP ADDRESSES HEALTH EXPOSURE	H-NAP ADDRESSES HEALTH EXPOSURE	IMPLEMENTATION PLANS AND MONITORING MENTIONED	INTERSECTORAL POLICY AND IMPLEMENTATION
The country is exposed to a wide range of hazards such as droughts, floods, and tropical cyclones	Direct risks to health posed by Climate change include risks with physical security; the potential exacerbation of the spread of diseases (such as malaria and cholera), and changes in patterns of respiratory and occupational diseases. Health impacts include under nutrition, trauma of displacement, water-borne diseases, and malaria (Arnall).	NAPA. 2007 No direct health specific initiatives included (Early warning; Strengthening agricultural production; impacts on coastal zones; water resources) Criteria used in prioritising NAPA and adaptation actions included Human health as a bullet in the “Poverty reduction and the increase of adaptation capacity”. This document identified that adaptation to climate change required a cross-sectoral approach, with the Ministry for the Coordination of Environmental Affairs responsible for coordinating climate change-related activities	H-NAP study conducted in 2019. This was a WHO assisted assessment of vulnerability and adaptation to climate change of the health sector in Mozambique. Health Sector Climate Change Adaptation Plan and finalization (expected November 2021).	National Climate Change Adaptation and Mitigation Strategy 2013–2025. Health 1 of 8 strategic areas. Includes the establishment of disease surveillance, food and water support programmes and increasing vulnerable people’s adaptive capacity through social protection systems. Mentioned the inclusion of a national level plan to implement the strategy and Local Adaptation Plans to support action at district level. By 2020, 86 local plans have been approved and 21 are under implementation (INDC 2020)	Adapting to Climate Change, GIZ 2012–2020 Mozambique Coastal City Adaptation Project, USAID 2014–2019 Cities and Climate Change–Pilot Program for Climate Resilience of Mozambique, World Bank. 2012–2019
Around a third of the Mozambican population live in coastal zones, with the low-lying human settlements and associated developments, as well as fishing activities, further vulnerable to severe flooding, sea-level rise, and associated stresses.	Changes in the geographic distribution and the prevalence in vector-borne diseases such as malaria Increased prevalence of water-borne diseases during flooding events, including diarrheal disease Less predictable disease transmission owing to the combination of variable rainfall and complex temperature changes (ADB/ACDI)		Developed National Health Observatory dashboard for climate sensitive diseases (Malaria, Dengue, Chikungunya, cholera, and diarrhoea) (WHO) The pilot for integration of climate and epidemiological information and development of early warning system in 4 provinces (Nampula, Sofala, Inhambane, and Maputo) is underway (no date).	Strengthen Reduce exposure to climate change vector disease. Strengthening capacity to prevent and control the spread of disease, including mapping of vector distribution and spatial mobility Establishing surveillance systems and control measures for specific diseases exacerbated by Climate Change	Climate Resilience: Transforming Hydro-Meteorological Services Project for Mozambique, World Bank. 2013–2019
	Food security	Health not specifically mentioned, but nutrition and food production noted		No mention of health intervention, but inclusion of agricultural resilience, agriculture, fisheries, food security and nutrition	

**Table 5:** Country-Level Data for Namibia Collated from the Full Data-Extraction Table [20].

CLIMATE	HEALTH	NAP ADDRESSES HEALTH EXPOSURE	H-NAP ADDRESSES HEALTH EXPOSURE	IMPLEMENTATION PLANS AND MONITORING MENTIONED	INTERSECTORAL POLICY AND FINANCING
Namibia has frequent dry-spells and droughts with sporadic occurrences of flooding in water basins. [Cuvelai drainage, Cubango-Okavango, Zambezi, Kunene, and Orange-Senqu rivers]. About 62% of Namibians live in rural areas and depend heavily on rain-fed agriculture, high number of female-headed households make Namibia even more vulnerable to impacts of climate change (NPCC)	Poor sanitary conditions due to floods in some areas, malnutrition due to reduced crop yields, and livestock productivity will increase illness and child mortality (NPCC) Increased prevalence of vector-borne diseases such as malaria and sleeping sickness, owing to better breeding conditions of mosquitoes and Tsetse flies, increased prevalence of water-borne diseases, such as cholera, increased potential for malnutrition and stunting owing to increased crop failure, increased number of people at risk of heat-stress (ADB)	-	-	The Namibia Vulnerability and Adaptation (V&A) Assessment report. 2008 Nominal mention of health (one paragraph in a p 167 document) National Policy on Climate Change 2011 Mention of heatstroke, respiratory disease, water- and vector-borne diseases amongst others. Health 1 of 19 strategies a) provision of safe water and sanitation facilities for the public (b) Provide medical assistance to the citizens of Namibia affected by climate change-induced diseases as well as malnutrition Mainstream climate change into the formal education system, at all levels Develop regional cooperation in order to deal properly with transboundary issues related to climate change (such as transboundary surface and ground water, transboundary disasters as well as regional climate change models) National Climate Change Strategy and Action Plan 2013–2020 created to implement Policy. (Support from UNDP, Gov of Japan, German Ministry of Econ Dev (GIZ) Development was a two-year process with multiple stakeholders and a focus on grassroots level to raise awareness and garner ideas from our local communities, who are most at risk to the impacts from climate change Health one of 4 priorities: (p 45) Strategic Aim 1: Health sector climate change strategy in place.	National Land Policy National Drought Policy Agriculture Policy SCORE Project (Scaling Up Community Resilience to Climate Variability and Climate Change) (2015–2019): climate-smart agriculture through micro-drip irrigation gardens and conservation agriculture through the enhancement of rain-fed crops. Most of the adaptation projects and programmes in Namibia are in the areas of energy, ecosystems, and agriculture. They focus on capacity-building, knowledge communication, field implementation, and policy formation and integration, with all nationally implemented projects supporting community-based adaptation. Africa Adaptation Programme in Namibia (2010–2012) Harambee Prosperity Plan
	Increased prevalence of vector-borne diseases such as malaria and sleeping sickness, owing to better breeding conditions of mosquitoes and tsetse flies Increased prevalence of water-borne diseases, such as cholera (ACDI)	-	-	Strategic Aim 2: Strengthen disease prevention and treatment for climate sensitive diseases. Adaptation strategies tailored to regions or communities based upon their risks and vulnerability Measuring and monitoring the effects of climate change on health will be very important. TARGET: By 2020 access to adequate health services specifically addressing climate related diseases increased by 80% (p 46)	75 million USD for the first three years: On-going improvement of sanitation done in conjunction with sanitation policy and strategy–more than half of the population in all towns have access to sanitation (NCCSAP)
It has been predicted with a high degree of certainty, that Namibia will become hotter throughout the year (NCCSAP)	Increased number of people at risk of heat-stress (ACDI) Increase in temperature will lead to heat-related illness and put to risk the already vulnerable groups such as the old, already sick people (e.g., with HIV/AIDS) and children (NCCSAP)	-	-	Strategic Aim 3: Develop adaptation mechanisms to climate change related health risks and disseminate information for effective preparedness. TARGET: Prevention and treatment measures strengthened, contingency plans developed and in place (p 46)	-
Maximum temperatures have been getting hotter over the past 40 years, as observed in the frequency of days exceeding 35°C, suggesting an overall warming (NCCSAP)	Increased potential for malnutrition and stunting owing to increased crop failure	-	-	Strategic Aim 4: Strengthen the existing mechanisms for the vulnerable groups to access basic services and health facilities during climate related emergencies. TARGET: (2014–2017)Put in place contingency plan to all vulnerable groups to access basic services during flood disaster periods b) Develop measures to safeguard vulnerable groups with emphasis on the special needs of women and children from flood and drought	-
**-**	-	-	-	Health impacts from Uranium dust (Erongo region)	-

**Table 6:** Country-Level Data for South Africa Collated from the Full Data-Extraction Table [20].

CLIMATE	HEALTH	NAP ADDRESSES HEALTH EXPOSURE	H-NAP ADDRESSES HEALTH EXPOSURE	IMPLEMENTATION PLANS AND MONITORING MENTIONED	INTERSECTORAL POLICY AND FINANCING
South Africa is a water-scarce country vulnerable to the effects of drought and rainfall variability (ACDI)	A significant proportion of South Africans, and in particular the poor, already have serious and complex health challenges compounded by poor living conditions The links between the environment, food security and infectious diseases profiles of communities and regions have been well established (NCCRP) These may lead to increased: malnutrition and negative implications for child growth and development deaths, disease, and injury due to heat waves, floods, storms, fires, and droughts burden of diarrhoea and respiratory diseases frequency of cardio-respiratory diseases due to higher concentrations of ground level ozone infectious disease carried by vectors water- and food-borne diseases vector- and rodent-borne diseases mental, nutritional, infectious, and other health effects (H-NAP)	South Africa’s National Climate Change Adaptation Strategy (2020) Health mentioned as one of six strategic priorities Areas include triple burden of disease, housing infrastructure, catastrophic events that impact health Lack of understanding of linkages between climate and health Four clusters to improve resilience and adaptive capacity including health actions, development of climate services with adaptation planning across all sectors, and national to local implementation. Education regarding climate change incorporated in to school and tertiary institutions Aim to set up an effective M&E system to track and assess success in achieving the strategic outcomes. Proposed indicators for each strategic outcome are shown	National Climate Change Health Adaptation Plan (2020–2024) Includes 9 priorities: Heat Stress Natural Climate hazards Housing and Settlements Communicable Diseases Exposure to Air Pollution and Respiratory Disease Non-communicable Diseases Vector- and Rodent-Borne Diseases Food Insecurity, Hunger, and Malnutrition Mental illness Components: National vulnerability and health system assessments (R100 000), capacity building, Integrated environment and health surveillance, research, monitoring, and evaluation Provinces and municipalities will be capacitated to undertake implementation Health sector mitigation measures are outlined in the Environmental Management Plan	National Climate Change Response Policy (2011) South Africa will, within two years of the publication of this policy, design and publish a draft Climate Change Response Monitoring and Evaluation System South Africa’s National Climate Change Adaptation Strategy (2020)	Climate Change Bill (2018) National Strategy for Sustainable Development (2011) National Environmental Health Policy (2013) The Department of Health Strategic Plan. National Development Plan (2030) Flagship Project with Long Term Adaptation Scenarios on climate change for sector departments Integrated Resource Plan 2019 National Environmental Health Norms and Standards for Premises and Acceptable Monitoring Standards for Environmental Health 2015
Under a high emissions scenario, mean annual temperature is projected to rise by about 5.1°C on average from 1990 to 2100.	Socio-economic standing of the most vulnerable communities, and the consequences in terms of food security and the nutritional status of individuals within these communities threatens to further undermine their resistance to diseases such as HIV/AIDS and tuberculosis.	Identify individuals (male and female) and communities at most risk from climate change within municipalities and deliver targeted climate change vulnerability reduction programmes for these individuals and communities	Response: Supplementary activities for response will be structured around the priority health risks as selected by the country. Comprehensive vulnerability and adaptation assessment conducted for all health risks categories considered priority at national level	Integrated nutrition programme (school health programme; primary health care approach programme, Ward Based Outreach Teams, the food for all programme, Reconstruction and development programme	-
Under a high emissions scenario heat-related deaths in the elderly (65+ years) are projected to increase to about 116 deaths per 100,000.	There is an increased number of people at risk of heat-related medical conditions, owing to increased temperatures	Identification of towns at high risk of heatwaves. Equip and capacitate emergency response departments, such as health and fire, to prepare for and manage climate related disasters	Develop intersectoral initiatives, employers/Department of Labour, capacity building	Mitigation: some District Health Services (DHS) are part of the Global Green Healthy Hospital Initiatives (GGHH) NCCHAP (p 18)	National Heat Health action guidelines WC Climate Change Response Implementation Framework (2014).
Higher temperatures and extreme rainfall (ACDI)	Increased prevalence of vector-borne diseases such as malaria Reduced water quality	Malaria early warning system The Infectious Diseases Early Warning System (IDEWS) project	Vulnerability and adaptation assessment established as an iterative process throughout the HNAP process (revised and updated periodically)	Strategic malaria control programme	South African Malaria Elimination Strategic Plan 2019–23
Extreme rainfall	Increased exposure to infections and physical injury in flooded or damaged areas owing to extreme rainfall	Adaptation plans for displaced people. Equip and capacitate emergency response departments, such as health and fire, to prepare for and manage climate related disasters	Improve/develop national early warning systems for key climate vulnerable sectors and risks	-	-
Air pollution	Respiratory diseases such as asthma, chronic obstructive pulmonary disease, allergic rhinitis, and bronchitis are most vulnerable, as are the elderly and young children	Improve resilience of ecological and physical infrastructure	Train national experts and environmental health fraternity on various climate change and health areas such as environmental sciences, epidemiology, public health, animal health, vector control, safety of drinking water, air pollution, sanitation, waste management, and soil degradation		National Environmental Management: Air Quality Act, 2004 National Framework for air quality management, 2017

**Table 7:** Country-Level Data for Zimbabwe Collated from the Full Data-Extraction Table [20].

CLIMATE	HEALTH	NAP ADDRESSES HEALTH EXPOSURE	H-NAP ADDRESSES HEALTH EXPOSURE	IMPLEMENTATION PLANS AND MONITORING MENTIONED	INTERSECTORAL POLICY AND FINANCING
The country is witnessing increased intensity of severe weather events, particularly droughts, prolonged intra-sessional dry spells, extreme storms associated with widespread hail and flash flooding, increasing incidences of heatwaves, and related health challenges These have disrupted the agricultural economy, hydroelectricity production, human settlements while also affecting critical communications and social infrastructure (NAP 2019) The economy of Zimbabwe is agro-based contributing about 15% each year to the GDP (NC3).	Highest mortality rates: HIV, TB, malaria, diarrhoea, child mortality and maternal health The major health issues in Zimbabwe relate to: Nutrition, water, sanitation and hygiene, health care services and facilities ante-natal and post-natal care. The country has a robust health information and surveillance report system.	NAP Roadmap, 2019 (Mentions National Strategy where health is one of 12 priorities) Climate Change Working Group: NAP working groups (3 pilot project areas) INC: Human health, vector-borne disease particularly malaria (will require investments in education and prevention techniques such as netting, repellents, and low-cost anti-malarial drugs.) Development of health care (NC3)	H-NAP exists but needs major revision for endorsement and implementation (WHO)	National Climate Policy 2017 1–6 adaptation areas Strengthen surveillance programmes for monitoring human health under a changing climate, particularly operational knowledge on climate-disease relationships Understand the impacts of climate change on women, children, youth, and people living with disabilities in Zimbabwe Undertake appropriate measures to fully realise the children’s rights to health in light of climate change Inclusion of Gender, Social Inclusion, and Climate Change National Climate Change Response Strategy 2014 Health one of 12 strategic areas Climate change education and training	Constrained by its limited ability to put in place appropriate measures to respond to climate change requirements because of lack of human, institutional, and financial resources GCF Readiness project, 2018. Climate-Smart Agricultural Investment Plan (CASAIP) The Response Policy has action plans and strategies which will require close to US 10 billion for implementation in the next 5 years, with 50% of the budget going toward water and agriculture related strategies National Gender response Plan (Health budget estimated 52 million USD)
The country relies on surface water resources for 90% of its requirements. In the urban areas, as much as 40% of water is lost during treatment and distribution	Increased prevalence of water-borne diseases, such as cholera, typhoid, and bilharzia, owing to intensified flooding (ACDI)			Develop and promote resilient water resources management Build resilience against diseases that occur because of impacts of climate change	
-	Changes in the geographic distribution of vector-borne diseases such as malaria, owing to changes in temperatures and precipitation (ACDI)	-	-	Enhance early warning and climate-related disaster risk reduction systems (including information management systems) a) Strengthen surveillance programmes for monitoring human health under a changing climate	Roll Back Malaria Campaign (WHO)
69% of the population are rural and reliant on smallholder subsistence farming and livestock. With a heavy reliance on rainfed agriculture and natural resources	The common nutritional deficiencies conditions were kwashiorkor, severe acute malnutrition, and pellagra	-	-	Develop, implement, and scale-up climate smart agriculture solutions and strengthen the resilience of agricultural value chains and markets	Food and Nutrition Security policy (2013) Considers risk reduction from climate change impacts. Now supported by the National Nutrition Strategy (2015)

The documentation available online varied for each of the five SADC countries (Table 1). Notably, only South Africa had submitted a NAP to the UNFCCC by 2021. The four remaining countries reported in their National Communications to the UNFCCC that they were in the process of submitting documentation but have various levels of national strategies, frameworks, and action plans in place (discussed in detail in the country-level sub-sections). While all five countries included health as a priority, only South Africa had completed a National Climate Change and Health Adaptation Plan by 2021. Botswana, Mozambique, Namibia, and Zimbabwe reported that H-NAPs were in progress, but no further documentation was found online. All five countries referenced having an adaptation policy and indicated that implementation plans were being developed.

## Summary of Study Countries’ Climate Hazard and Health Impacts

Climate hazard and health impacts from NAPs, strategies, and studies are summarized in Table 2. Common impacts include temperature changes leading to drought and flooding, with indirect health risks related to food and water insecurity. South Africa uniquely addressed mental stress and disease impacts.

## Country-Level Adaptation: Health Indicators, Policies, and Implementation Strategies

**Botswana:** With a population of two million, significant declines in rainfall (measured between 1970 and 2013) already have had a clear effect on the economy, human welfare, and the environment [25]. Botswana’s NAP Framework (2020) mentions that key sectors were included in a national consultation (no date provided), yet the Health Department was omitted. The Third National Communication (2019) highlights strategies for agriculture, maternal care, and environmental health, but despite the recommendations from the Second National Communication (2013) for the appropriate adaptation strategies required to address vulnerabilities, it lacks direct health-focused projects. This is shown in this review, which found that no studies focused on health impacts directly, and that only two studies addressed food security in Botswana (which would adversely impact population health because of malnutrition and related diseases) [26, 27].

Botswana’s Third National Communication (2019) identified limited country-based research that evaluated efforts to adapt to climate change, and the country’s NAP does not describe the relationship between future climate impacts and vector- and water-borne illnesses [25]. Stringer, examining policy-driven adaptation, notes that synergies between policy-driven and autonomous local initiatives are poorly developed [27]. The development of such links and the monitoring of programmes could be included in Botswana’s National Monitoring and Evaluation System outlined in the national strategy [28]. The national health impact assessments (not yet conducted within the study period) could also provide valuable information on population vulnerability.

**Mozambique:** With more than 29 million people, the country has a high vulnerability to climate events, due to its geographical location and scarce technical and financial resources [29]. Non-climate stressors include inadequate healthcare facilities and providers, high poverty levels, poor water supply and sanitation, food insecurity, and poor nutrition [30].

Mozambique’s Initial National Communication (2006) [29] indicates that health and fisheries were excluded in vulnerability assessments due to fiscal constraints. The NAPA (2007) included health only as a bullet point within the strategic priority area of ‘Poverty reduction and adaptation’ [29, 31].

Mozambique’s national strategy (2012) includes social protections and community-based adaptations for vulnerable groups, yet governance and accountability issues have delayed progress in these areas [33]. For instance, in 2016, Mozambique’s climate strategy coordination merged with the Ministry of Lands, Environment, and Rural Development amid performance and corruption issues [35]. It was under this merged ministry that Local Adaptation Plans were introduced and co-designed by districts and local communities. By 2020, 86 local plans were approved, with 21 under implementation (although details on health integration are unclear). No examples of the local plans were found online during the study period, yet it is likely that these plans were in Portuguese, which would not have been picked up in our search strategy.

Implementation strategies, such as the local climate adaptation plans, could be included in the monitoring and evaluation framework from the National Strategy [35]. The framework includes an adaptation indicator, measured through a household climate vulnerability survey, which included six health process indicators (details regarding these indicators were not outlined). Our online searches found no further survey data. Along with the monitoring frameworks, the National Strategy also highlights several barriers to effective implementation. These barriers include ineffective legal frameworks, inadequate data, limited knowledge, limited resources, and limited institutional capacity across national, district, and local levels [33].

Arnall’s case study on flooding and displacement in the Lower Zambezi River valley argues for a more political perspective to better understand the effects of resettlement and adaptation projects [32]. It highlights that Mozambique’s development of commercial agriculture and hydroelectric dams has significantly impacted community land use, income, and food security. This has caused significant changes in communities’, access to land, and income-generating activities, which place them at risk for increased poverty, mental illness, and food insecurity.

**Namibia:** With a population of two million people, Namibia has one of the lowest population densities in the world. It is also one of the driest countries in sub-Saharan Africa, with more than 20% of the country classified as desert and 70% classified as arid to semi-arid [36].

The National Policy on Climate Change (2011) and the National Climate Change Strategy and Action Plan (2013–2020) address health impacts but face barriers including limited capacity and fragmented implementation [37]. Importantly, the Strategy and Action Plans frame climate change as an environmental issue and highlight that insufficient evidence of the economic benefits of adaptation (versus a reactive approach) hinder efforts at implementation. As of 2022, Namibia had not submitted a NAP to the UNFCCC but had updated its NDC (2021) to include health adaptation priorities such as capacity building. Both the Fourth National Communication (2021) and its first Adaptation Communication (with technical support provided by the NAP Global Network) mention health adaptation.

**South Africa:** With a population of more than 55 million people, preventable disease conditions that are strongly associated with housing quality, such as diarrhoea and pneumonia, continue to be among the top-five causes of death among young South African children [38].

South Africa’s National Climate Change Adaptation Strategy (2020) follows a cluster approach and integrates health into various actions, including resilience-building and climate-sensitive health products (e.g., infectious disease early warning systems and identification of urban areas at high risk of heatwaves). The strategy includes a detailed monitoring and implementation framework with timeframes and indicators.

The H-NAP 2020–2024 lists 12 health priorities and vulnerability assessments (including heat stress, natural climate-related hazards, exposure to air pollution, and mental illness) [39]. A central component of that implementation is the inclusion of vulnerability and adaptation assessments conducted for all health risk categories that are considered a priority at the national level. Provincial-level climate action support and documentation are made available electronically through the ‘Local Government Climate Change Support Program,’ which includes a comprehensive toolkit for health sector vulnerability assessments [40].

Despite comprehensive planning, barriers include uneven resource distribution and implementation challenges. South Africa’s strategies and plans mention heightened national and regional vulnerability to climate change, exacerbated by limited access to healthcare, weak institutions, lack of appropriate skills and technology, and risk of armed conflict [39].

**Zimbabwe:** With a population of 13 million, poverty is estimated to affect around 63% [41]. It is a semi-arid country, with low annual rainfall reliability.

Zimbabwe’s NAP Roadmap (2019) lists health among its 12 priority areas [42]. However, the Health Department was not involved in the NAP assessment. The National Communications sent to the UNFCCC mention water, vector-borne diseases (especially malaria), and healthcare development. The 2014 National Climate Change Response Strategy aimed to enhance health surveillance and resilience to climate-related diseases, but specifics remained undefined as of 2022 [43]. In 2017, the National Climate Policy was finalised, and the National Gender Policy and Nutrition and Food Security Policy (2013) were amended to address climate change.

Barriers to implementation identified in the NAP Roadmap and UNFCCC communications include limited technical and institutional capacity, inadequate financial resources, insufficient climate data and monitoring, and uncoordinated efforts at various levels.

## Summary of Regional and Country-Level Governance and Strategic Plans

**Health Prioritization and Implementation:** Health is a priority in all five SADC countries’ NAPs. However, health departments were not consistently included in NAP assessments or in implementation plans. All countries have identified climate change focal points, often within environmental ministries, but they face challenges with governance and implementation across national, provincial, and local levels [40]. Mozambique and South Africa have local-level adaptation plans, but there is unequal development and resource allocation in these plans. For example, Mozambique’s early warning system project is limited to 4 of 11 provinces [34]. Geographic, political, and economic factors further affect program distribution and resource access [44].

**Sector Coordination:** Health impacts are often managed by sectors such as energy or agriculture, suggesting a need for better inter-ministerial coordination [27, 45]. South Africa has detailed cluster indicators and action points, while Namibia’s policy only briefly addresses health (in terms of safe water and medical assistance) [37]. Many countries’ health interventions remain siloed and under-resourced, with significant health impacts under-reported.

## Discussion

Despite the development of NAPs, health impacts are often addressed in isolation, leading to gaps between policy and practice. South Africa’s comprehensive H-NAP contrasts with that of other countries, where health impacts are less prioritized. Addressing the disconnect between policy scope and practice, including integrating health impacts of conflict and fragility, is crucial [46]. There is also a need to include economic benefits of adaptation in policy frameworks.

All five countries’ national adaptation strategies mention health alongside other priorities such as agriculture, energy, and water. However, only the impacts of food and water security and vector-borne diseases are consistently addressed in all five countries’ strategies. South Africa’s strategy uniquely includes air pollution and mental health impacts. Fragmented health systems and under-resourced departments are identified across the literature, but the increased demand on already-stretched health services and the disruption or collapse of health infrastructure and services due to extreme weather events is under-reported [23, 45]. National climate agendas could work to address the broader socio-economic determinants of health through integration into programming across sectors, strengthened stakeholder involvement, and measurable monitoring of activities and programmes [34, 47–50].

## Evaluation of National Strategies to Reduce Adverse Impacts of Climate Change on Health

National strategies provide a framework for programmes, but there is a need to strengthen evidence of implementation indicators and monitoring targets (e.g., timeframes, target groups, and measurable impacts). For example, Botswana’s NAP framework includes broad health interventions without specific indicators. The Third National Communication notes that Botswana’s Ministry of Health is not implementing climate-related health programs but does not explain why [25]. Comprehensive H-NAPs and health vulnerability assessments are needed to provide context-specific information for implementation indicators.

**Health impacts require greater prioritization:** Considering the significant burden of disease and fiscal resource constraints evident in the country reports, adaptation plans that integrate health across sectors can enhance resilience to climate change and address the challenges brought on by climate change. Currently, national strategies focus heavily on energy and agriculture, overshadowing health. For example, Zimbabwe’s Response Policy indicates that USD 10 billion is required for adaptation implementation, and yet 50% of the budget is directed towards water- and agriculture-related strategies [43]. Due to the region’s reliance on agriculture, this focus is not surprising, yet the near- and long-term effects of climate change across the sectors, including health, need greater attention.

**Governance, resource allocation, and monitoring remain uneven across countries:** Zimbabwe’s UNDP assessment (2021) found that climate policies could drive job creation if supported by social, labour market, and skills enhancement policies, all of which are lacking [41]. Similarly, Namibia identified climate change health indicators in its national policy (2011), but because its Health policy had not yet been revised, the implementation strategies are not yet in place [37].

**Development timelines for national climate and health plans are protracted:** Namibia, for instance, submitted its first UNFCCC National Communication in 2001. While its National Climate Policy was developed in 2011, it had not finalised its NAP more than 12 years after 2011. Zimbabwe’s timeline is also extended, with more than 20 years between its first communication and its policy development. Similarly, effective strategy implementation (which has the potential to reduce population vulnerability) has been delayed, as H-NAPs are still in process and as health policies have not been updated.

## Similarities and Differences in Implementation Approaches

**Sustainable Development and Climate Change:** All five SADC countries address climate change as a developmental issue, highlighting socio-political and economic impacts (such as infrastructure damage and access to food and health services), which disproportionately affect vulnerable populations. However, the health sector often remains under-prioritized and lacks integration across sectors, reducing the potential for comprehensive action [8].

**Health Impact Consideration:** Strategies acknowledge food and water security as significant threats (mentioned in all five strategies and addressed in 10 studies) but often overlook specific health impacts, such as maternal health and nutrition, which are exacerbated by inequitable access to food, water, and health services [51]. The shifting disease transmission paths and the complex relationships among climate, livelihood loss, and mental health are mentioned infrequently, with South Africa being an exception. A richer set of regional contextual studies is needed to address health risks comprehensively.

**Climate Information and Education:** All five countries’ plans emphasize climate information and education but lack details on methods and implementation. It is important to note that 80% of African youth have inadequate literacy skills, and, often, levels of vulnerability marginalise entry into education and adaptation opportunities [50]. The NAP emphasis on inclusion of youth and vulnerable groups in national programmes could go some way in addressing this skills deficit.

**Stakeholder Inclusion and Knowledge Integration:** While vulnerable groups are included in stakeholder meetings, these groups’ practical involvement in implementation plans and coordination remains unclear. Expanding the use of traditional, local, and community knowledge in NAP co-creation is essential to address this gap [52]. Monitoring progress reports on national strategies will help gauge ministerial commitment and evaluate stakeholder engagement and implementation [53–65].

## Opportunities to Strengthen SADC Coordination

**Multi-sector regional coordination:** This coordination offers distinct opportunities to address the lack of access to information, data, and limited institutional capacity [15] and can include analysing the adaptation funding flows (such as the UNFCCC SADC analysis between 2013 and –2017) [8] and improving SADC leadership of regional health diplomacy [17]. These steps could complement the continued framing, with a developmental lens, of climate change as an environmental issue.

**Accurate (in scale and frequency) climate data and mechanisms:** SADC member states have initiated efforts to enhance monitoring and development. Key initiatives include the Infectious Disease Warning Programme, Monitoring for Environment and Security in Africa, and Climate for Development in Africa, which aim to improve access to and use of earth observation data for policy development and collaboration. Updating SADC health frameworks and implementation plans could strengthen these synergies.

The study countries share **common climate and health impacts**, such as water and food insecurity issues and vector-borne diseases. Given the region’s geographic proximity among the countries and the interconnected socio-economic and political landscape, there are opportunities for regional cooperation to address these transboundary climate challenges. For example, Namibia’s Climate Change Policy highlights the need for regional cooperation on issues like transboundary water resources and extreme events [37]. Strengthened regional leadership could address cross-border impacts and mitigate the human and financial capacity deficits noted in the NAPs.

## Conclusion

The SADC region’s heavy reliance on agriculture and multiple stressors exacerbate the health impacts of climate change, including heightened food and water insecurity and the increased prevalence of water- and vector-borne diseases. Despite these challenges, health considerations are often under-prioritized, and implementation plans remain fragmented.

National Adaptation Plans (NAPs) and strategies need to be more agile and to incorporate measurable actions and timeframes to address health capacity and infrastructure deficits effectively. Strengthening governance structures and stakeholder engagement is crucial for enhancing health resilience. Additionally, regional leadership should coordinate efforts to address health-related climate issues, improve regional and national skills, and address resource gaps.

## Verification

All authors had access to the data and a role in writing the manuscript.

## Additional Files

The additional files for this article can be found as follows:

Supplementary File 1. Table A, Final search terms and criteria.

Supplementary File 2. Table B, National Level Reports identified from other sources.

Supplementary File 3. Table C, WHO indicator findings (WHO, 2021) for Mozambique, South Africa, and Zimbabwe.
