# Supplementary material for: Assessment of SADC Countries’ National Adaptation Planning Health Impacts Inclusion: A Thorough Review
Source: Ann Glob Health. 2024 Sep 18;90(1):57. doi: 10.5334/aogh.4458 (PMC11414460; doi:10.5334/aogh.4458)
Supplement: Supplementary File 1. — Table A, Final search terms and criteria. [file agh-90-1-4458-s1.pdf]

Supplementary material.

Table A: Final search terms and criteria

MeSH	KEYWORDS
	National Adaptation Plans OR NAP OR National Adaptation Strategy
	AND
"Climate Change"[Mesh]	Climate OR Climate change OR Global warming<br>OR Extreme weather OR Climatic Processes
	AND
"Health Impact Assessment"[Mesh] OR "Quality of Life"[Mesh]	Health OR Health system OR Health impact OR Health policy OR effect OR impact
	AND
"Africa, Southern"[Mesh]	Southern African Development Community OR SADC OR Southern Africa OR South Africa OR Botswana OR Namibia OR Zimbabwe OR Mozambique OR Angola OR Eswatini OR Lesotho OR Malawi OR Zambia OR Comoros OR Democratic Republic of Congo OR Seychelles OR Madagascar OR Tanzania OR Mauritius

Inclusion Criteria

The following inclusion criteria was used for identifying relevant publications:

i) Focused on one (or all) of the five-study country or countries within the SADC region.

ii) Included both climate change and human health impacts (direct and indirect pathways).

iii) Included NAP descriptions and / or national strategic responses to adaptation / climate change.

iv) Took cognisance of the NAP guidelines, with the technical guidelines expanded on by The Least Developed Countries expert group.

v) Publications were included irrespective of whether they were reviews, case studies or methodological articles.

Exclusion Criteria

The following exclusion criteria was used for not including publications:

i) Assessed or focused on countries in high-income countries.

ii) Assessed NAPs or national strategic responses to adaptation and climate change outside of the SADC region.

iii) Did not take cognisance of the UNFCCC NAP guidelines.

iv) Not written in English.
