# Supplementary material for: Assessment of SADC Countries’ National Adaptation Planning Health Impacts Inclusion: A Thorough Review
Source: Ann Glob Health. 2024 Sep 18;90(1):57. doi: 10.5334/aogh.4458 (PMC11414460; doi:10.5334/aogh.4458)
Supplement: Supplementary File 2. — Table B, National Level Reports identified from other sources. [file agh-90-1-4458-s2.pdf]

Supplementary material.

Table B. National Level Reports identified from other sources.

Botswana	National Adaptation Plan Framework for Botswana. 2020.
	Botswana Draft Climate Change Response Policy. 2016
	National Climate Change Strategy and Action Plan. 2018
	Initial National Communication to the UNFCCC. 2001
	Second National Communication to the UNFCCC. 2013
	Third National Communication to the UNFCCC. 2019
	Intended Nationally Determined Contribution to the UNFCCC. 2010
	Botswana. Vulnerability assessment committee results 2019.OCHA
	Climate and health country profile – 2015. Botswana. WHO
	Climate risk profile: Botswana (2021): World Bank Group
Mozambique	National Adaptation Programme of Action (NAPA) . 2007
	National Climate Change Adaptation and Mitigation Strategy 2013-2025. 2012
	Climate Change and Gender Action Plan for the Republic of Mozambique. 2014
	Meeting the challenges of climate change: monitoring Mozambique's National Climate Change Adaptation and Mitigation Strategy. Policy brief. 2016
	National Adaptation Plans in focus: lessons from Mozambique. UNDP. 2020
	Climate Change Adaptation in Mozambique . USAID
	Mozambique. National Climate Change Profile. ADB
	Intended National Determined Contribution to the UNFCCC. 2015
	First National Determined Contribution to the UNFCCC. 2018
	Update of the First Nationally Determined Contribution to the UNFCCC: 2020-2025<br>2021
	Initial National Communication to the UNFCCC. 2006
Namibia	National Climate Change Policy 2011
	National Climate Change Strategy and Action plan 2013 – 2020
	Namibia National Climate Change Profile. ADB
	Climate Change Vulnerability and Adaptation Assessment.2008 UNDP
	First Adaptation Communication to the UNFCCC. 2021.
	First National Communication to the UNFCCC. 2001
	Second National Communication to the UNFCCC. 2011
	Third National Communication to the UNFCCC. 2015
	Fourth National Communication to the UNFCCC. 2020
	National Determined Contribution update to the UNFCCC. 2021
South Africa	National Climate Change Adaptation Strategy. Republic of South Africa. 2021.
	National Climate Change Response Policy. Republic of South Africa 2011
	National Climate Change and Health Adaptation Plan. 2014-2019. 2014
	National Heat Health Action Guidelines. 2020
	Climate Change Adaptation Scenarios for Human Settlements. 2016
	Information sharing for adaptation planning at sub-national levels: South Africa's let's respond toolkit. 2017. NAP global network
	Climate and health country profile – 2015. South Africa. WHO

	Climate risk profile: South Africa 2021. World Bank Group
	South Africa National Climate Change Profile. ADB
	Western Cape Climate Change Response Implementation Framework. 2014
	Durban Climate Action Plan . 2019
	First Nationally Determined Contribution. 2021
	Recommendations on South Africa's draft updated Nationally Determined Contribution. 2021
	Initial National Communication. 2003
	Second National Communication. 2011
	Third National Communication. 2018
Zimbabwe	National Adaptation Plan (NAP) Roadmap 2019.
	National Climate Policy. 2017
	National Climate Change Response Strategy. 2014
	Nairobi H-NAP meeting report. 2018
	Climate Risk profile: Zimbabwe (2021); the World Bank Group
	Zimbabwe National Climate Change Profile. ADB
	Initial National Communication. 1998
	Second National Communication. 2013
	Third National Communication. 2017
	National Determined Contribution. 2021
