# Supplementary material for: Assessment of SADC Countries’ National Adaptation Planning Health Impacts Inclusion: A Thorough Review
Source: Ann Glob Health. 2024 Sep 18;90(1):57. doi: 10.5334/aogh.4458 (PMC11414460; doi:10.5334/aogh.4458)
Supplement: Supplementary File 3. — Table C, WHO indicator findings (WHO, 2021) for Mozambique, South Africa, and Zimbabwe. [file agh-90-1-4458-s3.pdf]

Supplementary material.

Table C. WHO indicator findings (WHO, 2021) for Mozambique, South Africa and Zimbabwe.

WHO Indicators	Mozambique	South Africa	Zimbabwe
Vulnerability and Assessment	Yes	In progress	No
NAP/ Strategy	Yes (roadmap)	In progress	Yes /No
Ministry Climate Change focal point	Yes	Yes	Yes
Climate Change task force	Yes	Yes	Unknown
Airborne and respiratory health surveillance.<br>Early Warning Systems	Yes<br>No	Yes<br>Yes	Yes<br>Unknown
Heat health surveillance.<br>Early Warning Systems	Yes<br>Yes	Yes<br>Yes	Unknown<br>Unknown
Injury/mortality EWE health surveillance.<br>Early Warning Systems	Yes<br>No	Yes<br>Yes	Unknown<br>Unknown
Malnutrition food borne disease health surveillance.<br>Early Warning Systems	Yes<br>No	Yes<br>Yes	Yes<br>Unknown
Mental and psychological health surveillance.<br>Early Warning Systems	Yes<br>No	Yes<br>Yes	Yes<br>Unknown
Vector borne disease health surveillance.<br>Early Warning Systems	Yes<br>No	Yes<br>Yes	Yes<br>Unknown
Waterborne diseases and other water-related health outcomes health surveillance.<br>Early Warning Systems	Yes<br>No	Yes<br>Yes	Yes<br>Unknown
Impacts on health care facilities	No	Yes	Unknown
Co -health benefits to climate change policy	No	Unknown	Yes
